# High road utilizers surveys compared to police data for road traffic crash hotspot localization in Rwanda and Sri Lanka

**DOI:** 10.1186/s12889-015-2609-1

**Published:** 2016-01-20

**Authors:** Catherine A. Staton, Vijitha De Silva, Elizabeth Krebs, Luciano Andrade, Stephen Rulisa, Badra Chandanie Mallawaarachchi, Kezhi Jin, Joao RicardoVissoci, Truls Østbye

**Affiliations:** 1 Division of Emergency Medicine, Duke University Medicine Center, Durham, USA; 2 Duke Global Health Institute, Durham, NC USA; 3 Department of Community Medicine; Faculty of Medicine, University of Ruhuna, Matara, Sri Lanka; 4 State University of West of Parana / Unioeste, Foz do Iguaçu, Brazil; 5 Public Health Research Group, Unioeste, Toledo, Brazil; 6 College of Medicine and Health Sciences, University of Rwanda, Butare, Rwanda; 7 University Teaching Hospital of Kigali, Kigali, Rwanda; 8 Southern Provincial Director of Health Services Office, Galle, Sri Lanka; 9 Department of Occupational Health; School of Public Health, Fudan University, Shanghai, China; 10 Faculty of Medicine, Faculdade de INGA, Maringa, Brazil

## Abstract

**Backgrond:**

Road traffic crashes (RTCs) are a leading cause of death. In low and middle income countries (LMIC) data to conduct hotspot analyses and safety audits are usually incomplete, poor quality, and not computerized. Police data are often limited, but there are no alternative gold standards. This project evaluates high road utilizer surveys as an alternative to police data to identify RTC hotspots.

**Methods:**

Retrospective police RTC data was compared to prospective data from high road utilizer surveys regarding dangerous road locations. Spatial analysis using geographic information systems was used to map dangerous locations and identify RTC hotspots. We assessed agreement (Cohen’s Kappa), sensitivity/specificity, and cost differences.

**Results:**

In Rwanda police data identified 1866 RTC locations from 2589 records while surveys identified 1264 locations from 602 surveys. In Sri Lanka, police data identified 721 RTC locations from 752 records while survey data found 3000 locations from 300 surveys. There was high agreement (97 %, 83 %) and kappa (0.60, 0.60) for Rwanda and Sri Lanka respectively. Sensitivity and specificity are 92 % and 95 % for Rwanda and 74 % and 93 % for Sri Lanka. The cost per crash location identified was $2.88 for police and $2.75 for survey data in Rwanda and $2.75 for police and $1.21 for survey data in Sri Lanka.

**Conclusion:**

Surveys to locate RTC hotspots have high sensitivity and specificity compared to police data. Therefore, surveys can be a viable, inexpensive, and rapid alternative to the use of police data in LMIC.

**Electronic supplementary material:**

The online version of this article (doi:10.1186/s12889-015-2609-1) contains supplementary material, which is available to authorized users.

## Background

Over 1.24 million people die annually on the world’s roads, with another 20 to 50 million sustaining non fatal injuries due to road traffic crashes (RTC), and these numbers are increasing rapidly [1]. Low and middle income countries (LMIC) are facing most of this burden with almost three times higher rates of road traffic injuries compared to high income countries [1]. Globally, half of all traffic deaths are amongst vulnerable road users: pedestrians, cyclists and motorcyclists [2]. Ultimately, the burden of road traffic injuries rests mainly upon vulnerable road users in LMIC where safety standards are limited and health systems are immature.

The World Health Organization (WHO) recommends that existing road infrastructure should be assessed for safety at regular intervals with a focus on roads with the highest crash risk [1]. Given the high cost of assessing all roads, focused safety assessments on locations where road traffic crashes, injuries, or deaths have occurred is more financially feasible. The WHO suggests that best practice road safety audits should include an assessment of safety for all road users, including pedestrians, cyclists, and motorcyclists [1]. To conduct these road safety audits complete epidemiological data, including crash locations, is required. While most countries utilize police, prehospital, or hospital based data to identify road traffic injury hotspots LMIC data sources may have incomplete and poor quality data. In particular they may lack latitudinal or longitudinal data or not have addresses amenable to subsequent determination of geolocation coordinates [3–6]. Many have proposed linking datasets from police and hospital systems to reduce these limitations; however this option can be costly, require strong hospital and police infrastructures, and still can have upwards of 40 % missing data for seriously injured patients even in high income countries [5, 7–9].

Given the previously mentioned challenges, there are no generally accepted alternatives to police or hospital datasets to inform RTC hotspot analyses in LMIC settings. In order to evaluate a timely, complete, low cost, and easily available data source to inform RTC hotspots for road safety assessments, this project compares a survey-based questionnaire of high road utilizers to police data for identification of RTC hotspots in two country settings, Rwanda and Sri Lanka. Kigali, Rwanda’s capital and home to just over 1 million people, was the focus of this study [10]. In 2012, there were 4471 RTCs throughout Rwanda, 80 % of which occurred in Kigali [11, 12]. In Sri Lanka, we focused on the Galle Municipality within Galle District in southern Sri Lanka with a population of 101,159 people [13]. The most recent national data estimates in Sri Lanka suggest that in 2005, there were approximately 2300 deaths and over 300,000 non-fatal injuries due to RTC [14]. We specifically choose a group of vulnerable road users in each country who were the most frequent road utilizers, as they would be the most aware of dangerous locations especially from a vulnerable road user perspective. As both of these environments have a significant burden of injury amongst vulnerable road users, these populations were chosen to mirror that burden and provide the vulnerable road user's perspective.

While police datasets are known for underreporting and underestimation, there is no gold standard alternative. As such, the purpose of this study is to evaluate a survey of high road utilizer users and determine whether this survey method is comparable to police data. We hypothesized that surveying vulnerable road users who are high road user utilizers could identify and locate RTC hotspots at least as well as police data. Vulnerable road users were chosen as they have a unique perspective and suffer the largest burden of injury. We secondarily calculate the marginal cost of conducting such surveys.

## Methods

### Ethics

This project was approved by the Central Hospital University of Kigali (CHUK) Ethical Committee; Rwandan National Ethics Committee; the Ethical Committee of Faculty of Medicine, University of Ruhuna, Galle, Sri Lanka; and the Institutional Review Board of Duke University in Durham, North Carolina. In both Rwanda and Sri Lanka, we obtained permission and fostered collaborations with the local police in order to conduct the project.

### Study settings

In Rwanda, this project focused on Kigali, which is 730 km^2^ in size and home to just over 1 million people [10]. The population density of Kigali has risen from 1049 people per km^2^ in 2002 to 1556 people/sq km in 2012. In total in Rwanda, there are 4700 km of roadways of which only 1207 km are paved and there were 140,149 vehicles registered in 2013. In 2012, there were 4471 RTCs throughout Rwanda, 80 % of which occurred in Kigali [11, 12].

In Sri Lanka, this project focused on the Galle Municipality within the Galle District in southern Sri Lanka which has a population of 101,159 people in 18.7 km^2^ [13]. Galle District had a population density of 658 persons/ km^2^ in 2012. In total, in Sri Lanka there are 114, 093 km of roads of which only 16,977 km are paved. In Sri Lanka in 2014, there were 211, 979 total registered vehicles of which 44,876 were three-wheelers; in Galle specifically, there were 28,701 total vehicles of which 7152 were three-wheelers and 20,421 are motorcycles. The most recent national data estimates in Sri Lanka suggest that in 2005, there were approximately 2300 deaths and over 300,000 non-fatal injuries due to RTC [14].

The two study countries were specifically chosen to represent different continents, different populations (dense urban versus urban), and different police reporting infrastructures; Sri Lanka has a legal non-reporting mechanism where crashes can be not reported and road side agreements made between parties legally for non-fatal injuries whereas Rwanda does not [3]. Given that police data in these two locations is not formally analyzed or distributed, we anticipated that our surveyed population did not have any familiarity with the retrospective police data.

### Data collection

#### Police data collection

Retrospective data was collected from the police datasets from each setting. Information available included the crash logistics, locations of the crash, involved persons, and severity of injury. Geolocation coordinates were determined based on addresses and description in police data, or the latitude and longitude if available. These were entered for each description of RTC location for further spatial analysis. The severity of injury in Sri Lanka was labeled as no injuries, non-grievous injury, grievous injury, and fatality while in Rwanda injuries were listed as fatal, grievous, and non-grievous. These categorizations were determined by police according to their police investigation with input from any treating practitioner, if available, but validity and inter-rater agreement amongst these categorizations are untested. As such, we further categorized these into fatal/grievous or non-grievous/no injury to limit any possible bias. While neither Kigali, Rwanda nor Galle, Sri Lanka had apriori studies evaluating their completeness Kandy, Sri Lanka had been found to have a 33–56 % estimated rate of underreporting based on a capture-recapture community survey and police data follow-up [3].

#### Survey data collection

Trained research assistants conducted pilot surveys amongst the high road utilizer population in each country. They utilized electronic data entry and tablet computers for direct data entry into an online database. Questionnaires included respondent demographics such as work history and amount of time spent on roads. Respondents were questioned on their knowledge of the research area in question to ensure they were high road utilizers of the research area. They were asked to identify dangerous locations and label the severity of danger of each location on a 0–100 scale. The survey in its electronic form was pilot-tested in each location to ensure appropriate translation, comprehension of questions, and electronic data management capacity. Based on pilot testing, question wording, and translation were improved upon to improve the questionnaire understandability and improve responses.

The research team in Sri Lanka identified 300 tuk-tuk (three wheel) drivers total from each sector of Galle to ensure spatial representation of the municipality. A tuk-tuk stand located in each sector was chosen at random and visited at a random time during the day by a trained Sri Lankan research assistant. Tuk-tuk drivers were approached and offered participation in the study after informed consent and were reimbursed the cost of an average tuk-tuk fare for their time ($1.50 USD). In Rwanda, the national moto (motorcycle taxi) driver association formally designates stands to provide service in the most populated locations across Kigali. These stands were chosen across Kigali to ensure spatial representation of the city. At each of these stands the first ten moto drivers encountered were offered survey participation by trained, native-Kinyarwanda speaking research assistants who conducted the surveys in Kinyarwanda after informed consent . Surveys were intentionally brief to maximize participation and minimize potential loss of income; moto drivers were reimbursed at about the average cost of a ride for their time participating in the research ($0.68USD).

#### Differences in data collection methods

Differences in data collection between Sri Lanka and Rwanda were determined based on pre-testing results, respondents’ comfort with the questions as well as applicability of the questions. The ‘dangerousness scale’ was not utilized in Rwanda data due to logistical difficulties with the scale using the computer-based data entry but was successfully used in Sri Lanka.

#### Data management

Study data was collected directly into and managed using REDCap hosted by Duke University [15]. REDCap (Research Electronic Data Capture) is a secure, web-based application designed to support data capture for research studies, providing 1) an intuitive interface for validated data entry; 2) audit trails for tracking data manipulation and export procedures; 3) automated export procedures for seamless data downloads to common statistical packages; 4) procedures for importing data from external sources and 5) secure storage.

#### Data analysis

To assess the data collected through the surveys, we used RTC data from police as the criterion standard. While police data is known to have extensive underreporting, most LMIC do not have prehospital care nor extensive hospital-based datasets that include RTC location information [6, 16–21]. Thus, police data remain the best available sources for RTC location.

We evaluated RTC locations for both Galle, Sri Lanka and Kigali, Rwanda by evaluating for clusters, or hotspots, of RTC geographically. A hotspot, for this project, is an area of high density of road crash locations. Our analysis plan was comprised of four steps. First, RTC locations were mapped into polygons; then, each polygon was classified by risk (low, medium, high) based on the density of occurrences within that polygon. Using these classifications, we then conducted an agreement and association analysis between methods. Finally, to compare our survey method to police data, we calculated sensitivity and specificity of the survey relative to the police data, adopting two risk classification outcomes (low and medium/high risk).

#### Spatial analysis

Spatial analysis was used to geographically localize RTC locations and identify specific distribution patterns through cartographic visualization [22, 23]. The georeferenced cartographic database with Political-Administrative Division of the Kigali Province (3 districts - Gasabo, Kikukiro, Nyarugenge) is freely available online in SHP format (shapefile) at the National Statistics Office of Rwanda (http://www.statistics.gov.rw/geodata), and the cartographic database of Galle was available from the Department of Census and Statistics of Sri Lanka (http://www.statistics.gov.lk/). We applied a kernel density estimator to verify these spatial patterns. This estimator establishes a two-dimensional function of location, forming a surface whose value is proportional to the intensity of samples per unit of area, so-called “hotspots” [22–24]. This function performs a count of all locations within a region of influence, weighting them by the distance of each point to the location of interest. We utilized a weighted measure to increase the influence of crash severity on the resulting “heatmap”; for survey data, we used the survey respondent ranking of dangerousness (0–100), and for the police dataset we used the severity of the crash (no injury, minor injury, severe injury, fatal injury). For spatial analysis we used Quantum GIS (QGIS) version 2.2.0 - Valmiera [QGIS] [25].

#### Agreement analysis, sensitivity and specificity

To provide the same metric for comparison for both police data and survey data spatial distribution points, we applied a vectoral grid disposition of polygons to maps (QGIS) after KDE, building a map based on the same number of polygon for both methods of data collection. An average polygon KDE was calculated based on individual point’s KDEs clustered within each analysis unit (polygon). Based on the average KDE, we then classified each polygon into a Low, Medium and High risk for RTC considering previously explained spatial distribution cutoff points for hotspots analysis.

Agreement between police and survey spatial polygon risk classification was conducted using percent agreement and Cohen’s Kappa. To calculate the confidence intervals for Kappa values we used a bootstrapping method based on a 1000 randomized samples. Values above 0.40 were considered moderate; values above 0.60 were considered high correlations. Sensitivity and specificity were calculated for survey data hotspots relative to police dataset-determined hotspots. All analyses were performed using R Language [26].

#### Cost analysis

A cost comparison was conducted for the data collection method in each country. Costs included research personnel, equipment, and transportation costs. Police data personnel costs do not include costs of police personnel who collect, enter, accumulate, or distribute this data to researchers but does include costs for data entry personnel who managed research data. Other costs include cost of incentives given to tuk-tuk drivers for participating and cost of food and refreshments for participants and data collectors. The cost per method was calculated per crash location determined as it is this data point which allows for power in our analysis.

## Results

In Rwanda 3191 records were included, with 2589 from police data and 602 from surveys. Rwandan police data entries were able to identify the crash location 72 % of the time. In Sri Lanka, a total of 1052 records were included, with 752 from police and 300 from surveys. Sri Lankan police data had a high location identification at 96 % of records. Characteristics of the Sri Lankan and Rwandan datasets are listed in Table 1.

**Table 1:** Characteristics of Rwanda and Sri Lanka survey and police data

	Kigali		Sri Lanka	
	(730 km2, 1 million people)		(18.7 km2, 101,159 people)	
	Police	Survey	Police	Survey
# Responses	2589	602	752	300
# Locations	1866	1264	721	3000
% Male (n)	Driver 1: 91.9 % (2376)	100 % (602)	90 % (673)	99 % (298)
	Driver 2: 81.2 % (1873)			
Mean Age Injured Person (sd, range)	35.2 (±9.1, 16–74)	N/A	39.5 (±13.9, 3–87)	N/A
Mean Age Surveyed Person (sd, range)	N/A	30.1 (±6.4, 18–57)	N/A	41.3 (±9.8, 19–68)
Mean Hours worked per week (sd, range)	N/A	76.5 (±15.2, 6–140)	N/A	75.9 (±18.6, 28–128)
Mean year in this profession	N/A	5.4 (±4.1, 0–28)	N/A	10.7 (±10.7, 1–32)

**Fig. 1:**
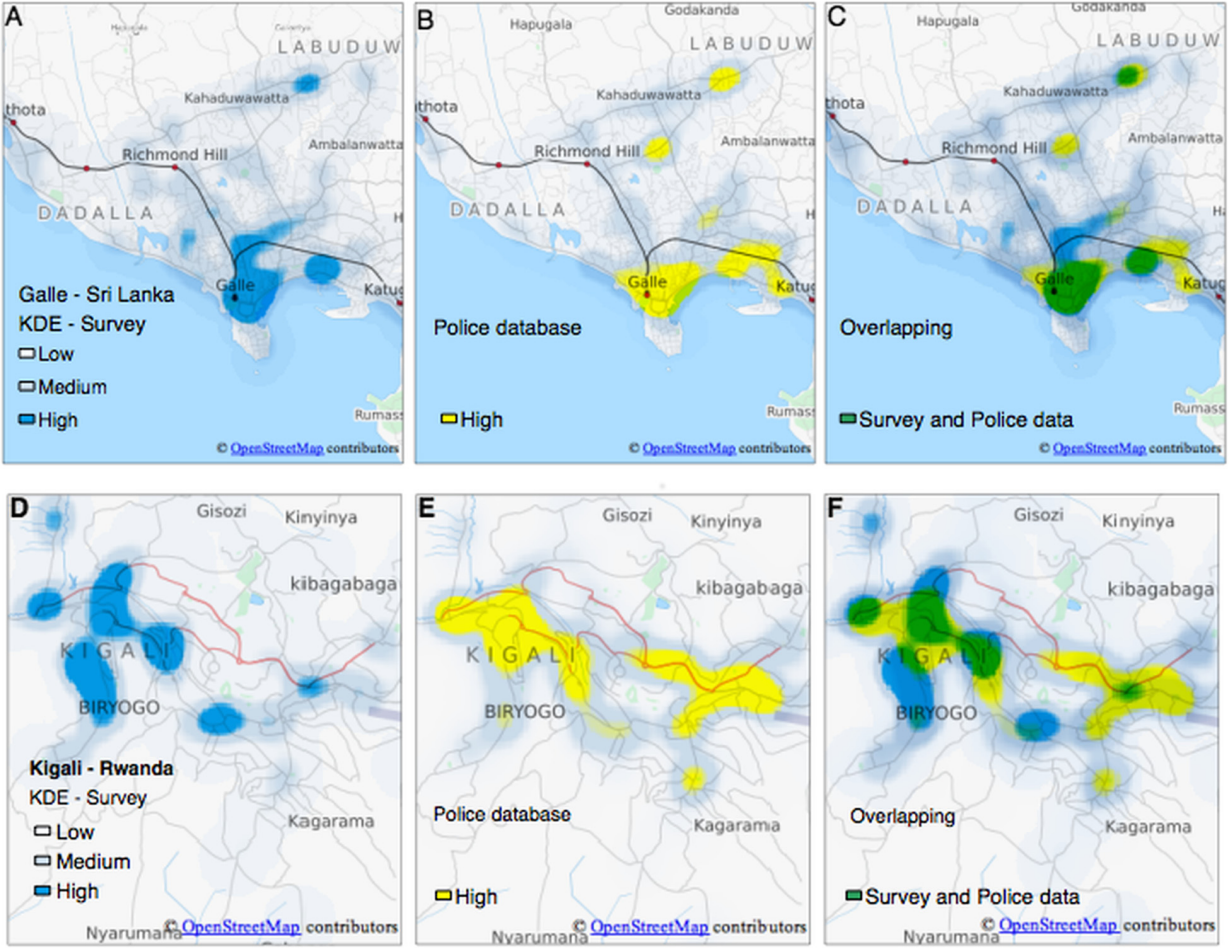
Kernel Density Estimation of survey and police data derived RTC hotspots weighted by severity of injury (**a**,**b**,**c**,**d**,**e**,**f**)

We mapped hotspots of RTC based on survey data and police data for Kigali, Rwanda (Fig. 1a, b) and Galle, Sri Lanka (Fig. 1d, e). By overlapping the survey and police data as seen in Fig. 1c and f for Kigali, Rwanda and Galle, Sri Lanka, respectively, visual representations emerge of locations identified by police data only, survey data only or both data sources.

### Agreement and associations

**Fig. 2:**
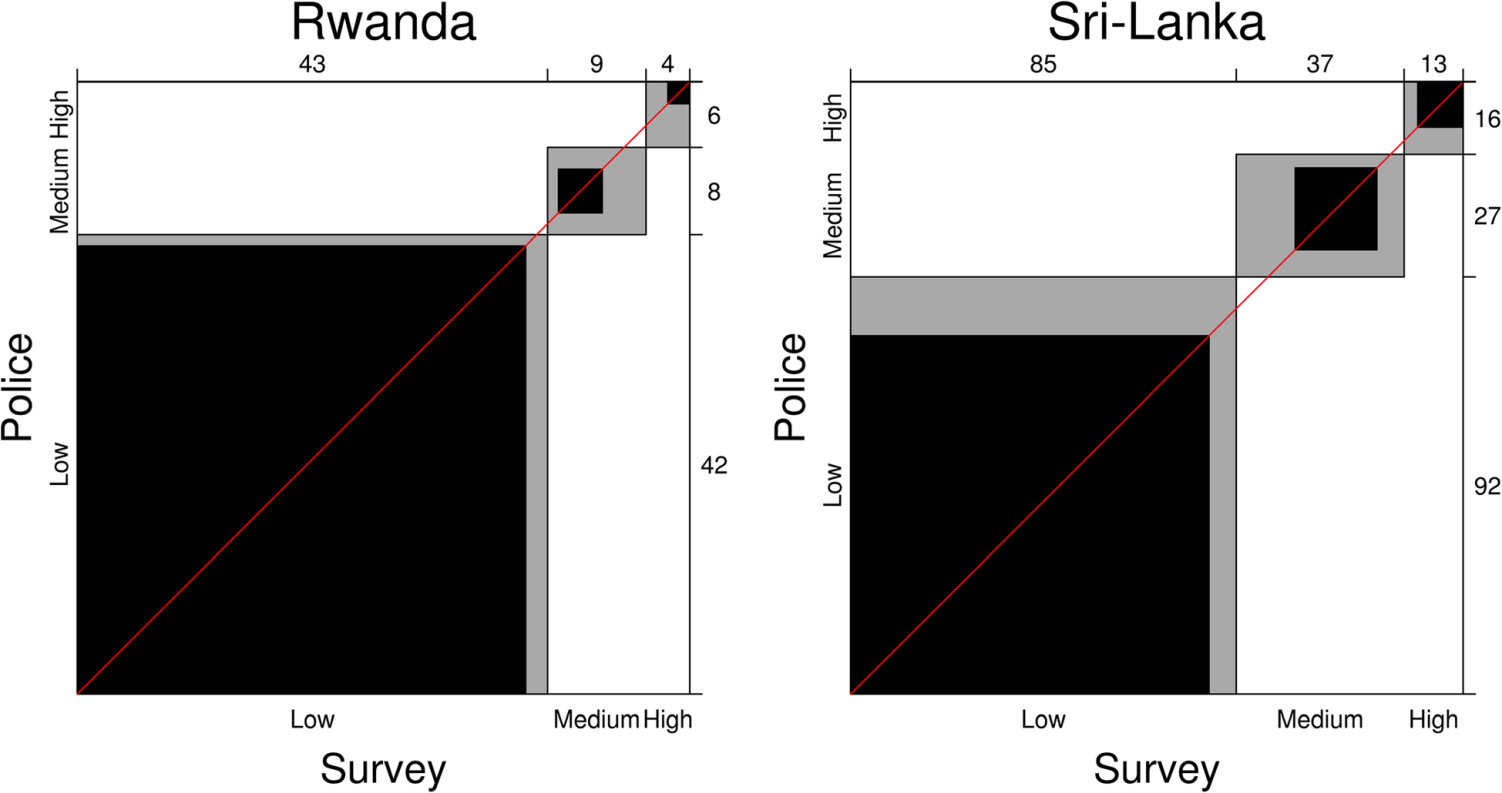
Agreement of polygons of Low, Medium and High risk for crashes for Survey compared to Police Data in Rwanda and Sri Lanka. Frequencies of polygons classified in each strata (*Low, Medium, High*) are listed on top and side. Black areas illustrate agreement between both methods of data collection while grey areas represent lack of agreement

**Fig. 3:**
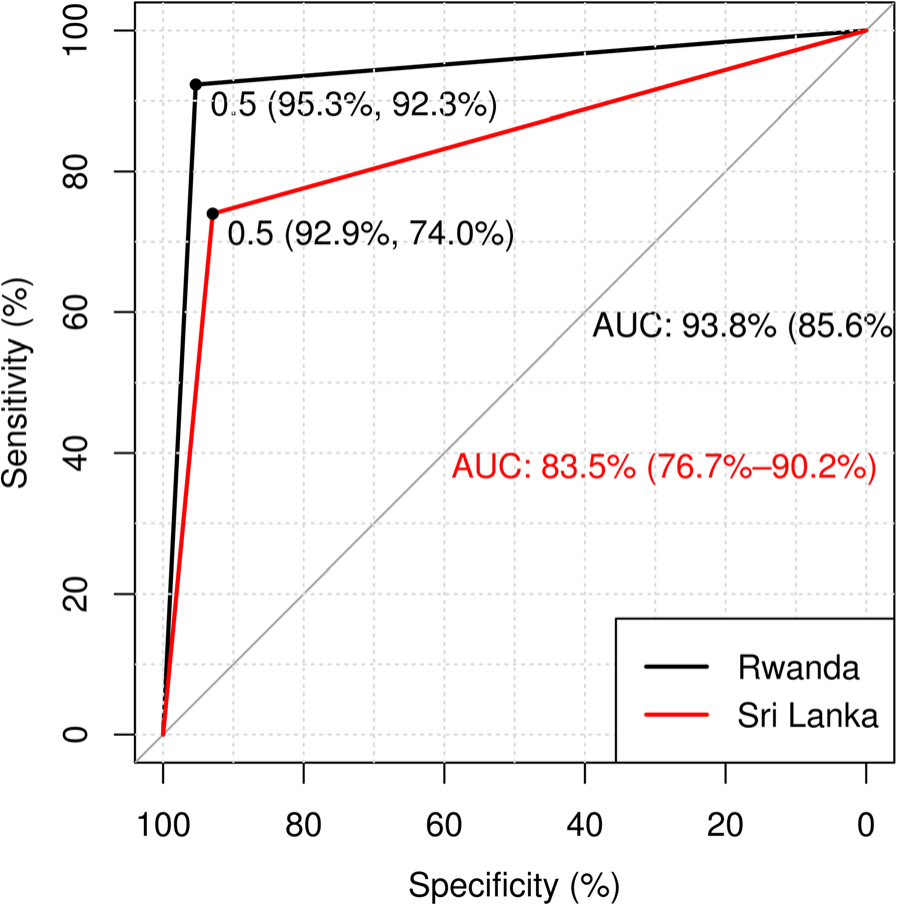
Sensitivity and Specificity and Area under the Curve (*AUC*) for Survey compared to Police Data in Rwanda and Sri Lanka

The police and the survey data showed a high agreement in both countries –83.9 % in Rwanda and 79.3 % in Sri Lanka. The Cohen’s Kappa was 0.80 (high, 0.69–0.90 and 0.69–0.86, respectively) for both Rwanda and Sri Lanka datasets, and AC1 correction was also high (0.79), illustrated in Fig. 2. The sensitivity and specificity of survey data for Rwanda was 92.3 and 95.3 with an AUC of 93.5 % (85.6; 100.0), respectively, while for Sri Lanka they were 74.0 and 92.9 respectively with an AUC of 83.5 % (76.7; 90.2). The ROC curves for Rwanda and Sri Lanka are displayed in Fig. 3.

### Project costs

Total costs of this project for personnel, transportation, and equipment by country and by data collection method are listed in Table 2. The cost per crash location identified as calculated as $1.21 compared to $2.75 in Sri Lanka and $2.75 compared to $2.88 in Rwanda.

**Table 2:** Relative costs of survey and police data in Rwanda and Sri Lanka

	Rwanda		Sri Lanka	
Costs (USD)	Police data	Survey data	Police data	Survey data
Personnel	4373.00	2648.00	1230.77	2000.00
Transportation	448.00	429.00	153.85	384.62
Equipment	225.00	200.00	561.54	561.54
Other	327.00	200.00	38.46	692.31
Total	$5373.00	$3477.00	$1984.62	$3638.46
Cost/Crash Location	$2.88	$2.75	$2.75	$1.21

## Discussion

The objective of this project was to evaluate a survey of high road utilizers to inform RTC hotspots in comparison to the more commonly used, but at times limited, police data. To our knowledge, this is the first project to identify an alternative survey method of RTC hotspot identification. Our survey utilized crowdsourcing methodolgy and was designed to be reproducible, cost-effective, and generalizable in low and middle income countries.  It should be noted that police records serve a primarily legal function; their use for research and policymaking is secondary. While superior quality police records would be optimal, this is not feasible in many LMIC. As such, alternative or adjunct methods are warranted. Our high road utilizer survey had a high agreement (84 %, 79 %) and kappa (0.80) with a good sensitivity (92 %, 74 %) and specificity (95 %, 93 %) for Rwanda and Sri Lanka, respectively.

We further demonstrated that survey data could be collected at low cost. In contrast to police records, which rely on passive data collection, surveying allows active data collection. This active approach allows for data collection in areas of interest to provide enriched, relevant information. Incomplete agreement between the methods suggests that surveys can act as an adjunct to existing police records by identifying crash locations missed by standard police data. Finally, surveys provide a medium for high road utilizers to report dangerous traffic spots. This user engagement can have significant benefits for LMIC policymakers and administration. Overall, our high agreement and high sensitivity results suggest that survey-based data collection of high road utilizers is a feasible low - cost alternative for researchers and policy makers in settings where police data might have quality or completeness limitations.

According to the World Bank, Rwanda had one of the worst road-safety records in 1996. But after implementation of a Rwandan government /WHO Road Safety program, there have been marked improvements- laws enforcing helmet use, penalizing drunk-driving and speed and road user education have reduced road deaths by 30 % [27]. In Rwanda, given the large area and dense population, we attempted to include more police and survey records in order to adequately survey this larger population. Unfortunately, but not unexpectedly, police data often did not include latitude or longitude or a description of the location in such detail that the RTC location could be located on a map. Only 72 % of the police records could be used for the mapping and hotspot analysis portions of this project. It is common to have these limitations in mortuary, police, and hospital data; while prehospital care records sometimes include this information, they suffer the same quality limitations that can been seen in other datasets [4–6, 28, 29].

In contrast, Sri Lanka has had a limited national response to road safety prevention even though there have been multiple calls for action [14, 30]. There appears to be a large underreporting of RTCs in Sri Lanka with estimates of 33–56 % of crashes not being reported in police data, including severe and fatal injuries [3, 14]. Of even greater concern, recent ‘on the spot’ insurance and insurance premium cost reductions for not being involved in a crash allow RTC to be financially settled between participants without police report, which is likely to lead to further underreporting [3].

When comparing both Sri Lankan and Rwandan experiences, methodological differences might have accounted for the difference in sensitivity and specificity. Sri Lanka had a higher sampling per density than Rwanda and had 10 locations identified per survey compared to Rwanda’s 1–2 location per survey. These two differences could have greatly decreased the sensitivity of the survey methods.

The overall costs for surveys in Sri Lanka were greater than for police data collection for the survey. Since the power of geographic information systems rests on the number of locations in order to determine hotspots, we calculated the cost per dangerous location (survey) or RTC location (police) identified. Per location identified, the cost was lower for both Rwanda and Sri Lanka. While each of the hotspots identified will need further evaluation in order to suggest a public health improvement and its associated cost, there is a reduced cost of identifying RTC hotspots using this survey method.

The WHO has suggested the development of global road safety assessment standards [2]. To achieve appropriate hotspot identification, we have developed, and tested a low cost survey method which is easily reproducible and interpretable. This new survey method is an easily replicated road safety research tool available at a low cost. It has been piloted in two countries and is generalizable to low and middle income countries, where these audits are seldom performed due limited data availability.

### Limitations

There are some limitations to this project which must be acknowledged. First, there is no true gold standard for determining RTC locations. Both countries are likely to have underreporting; rates of underreporting are highly variable by city and region. Rwanda has no documented underreporting rates currently, and Sri Lanka has cited a rate of approximatley 33 %. [3]. Therefore, the impact of underreporting could have a differential impact on survey performance outcomes between Rwanda and Sri Lanka. However, police records have been adopted as the most available and utilized option and as such, they were used in order to show how well the current survey performs against this standard. Given these difficulties, we performed this survey in two different locations in order to show generalizability, compare and improve methods, and suggest potential improvements based on location differences. During our project, the same people who were reading descriptions of the police data and determining the RTC location were doing the same for the survey data. While there might be some contamination, these locations were determined at separate time intervals from each other in order to reduce this potential bias. We anticipated that identification of locations are likely to be at the level of an intersection or road segment and therefore used spatial analysis to account for small differences in latitude and longitude reporting.

## Conclusion

Low and middle income countries carry a large and increasing burden of road traffic injuries and have limited ability to perform road safety assessments as suggested by WHO. Our survey method offers a low cost alternative that is not inferior to and may contribute Additional file 1. Surveying high road utilizers can be a valid, inexpensive, and rapid alternative to the use of police data in low and middle income settings where police data might be limited.

## Additional file

Additional file 1:
**High Road Utilizers Surveys Compared to Police Data for Road Traffic Crash Hotspot Localization in Rwanda and Sri Lanka.** (DOCX 28 kb)

## Abbreviations

CHUK: Central Hospital University of Kigali
KDE: Kernal density estimation
LMIC: Low and middle income countries
RTC: Road traffic crashes
WHO: World Health Organization
