# Supplementary material for: High road utilizers surveys compared to police data for road traffic crash hotspot localization in Rwanda and Sri Lanka
Source: BMC Public Health. 2016 Jan 20;16:53. doi: 10.1186/s12889-015-2609-1 (PMC4719689; doi:10.1186/s12889-015-2609-1)
Supplement: Additional file 1: — High Road Utilizers Surveys Compared to Police Data for Road Traffic Crash Hotspot Localization in Rwanda and Sri Lanka. (DOCX 28 kb) [file 12889_2015_2609_MOESM1_ESM.docx]

**Author’s Response To Reviewers**

**Title:** High Road Utilizers Surveys Compared to Police Data for Road Traffic Crash Hotspot Localization in Rwanda and Sri Lanka

**Authors**

Catherine A. Staton (catherine.staton@duke.edu)

Vijitha De Silva (pvdesilva@gmail.com)

Elizabeth Krebs (krebse@gmail.com)

Luciano Andrade (luc.and1973@gmail.com)

Stephen Rulisa (s.rulisa@gmail.com)

Badra Chandanie Mallawaarachchi (chandipushpa@yahoo.com)

Kezhi Jin (zhkjin@fudan.edu.cn)

Joao RicardoVissoci (joaovissoci@gmail.com)

Truls Østbye (Truls.ostbye@dm.duke.edu)

**Version:** 2 **Date:** 21 October 2015

**Author’s Response to Reviewers:** See over

**Reviewer’s Report**

**Title**: High Road Utilizers Surveys Compared to Police Data for Road Traffic Crash Hotspot Localization in Rwanda and Sri Lanka

**Version**: 2 **Date**: 31 July 2015

**Reviewer**: Davoud Khorasani-Zavareh

**Reviewer's report:**

First of all thanks authors well written manuscript. It is interesting manuscript,

however, the major concern for me on this study is that approximately in all

country police data suffer from under reporting and under estimation, which in

this study didn't consider that when they compare by survey based study. and

they recommend a survey instead of current work of police that cannot be

acceptable and needs more caution. The other comments are on the PDF file.

---------------------------------------------------------------------------------------------------------

Comments from PDF File With Responses:

1. Lines 118-121: One of cost effective method to overcome this is capture recapture method. Why you don't relay on that and why you don't talk about that?

- Capture- recapture has been used as a very successful method of determining rates of under reporting and estimating 'missingness' in a data set but unfortunately, this method will not assist in providing data that is not routinely included in some LMIC datasets like latitude and longitude and addresses which would allow geolocation coordinates.

1. Major concern for me on this study is that approximately in all country police data suffer from under reporting and under estimation, which in this study didn't consider that when they compare by survey based study. and they recommend a survey instead of current work of police that cannot be acceptable and needs more caution

- This is a great point and the basis for this project. Knowing that there are major concerns with underreporting and under estimation for police data sets, we were searching for a non-inferior method. As there is no true 'gold standard' the goal of this project was to show that using a survey such as our is 'NOT INFERIOR' to using police data especially when police data might be quite limited.

1. Lines 123-124: what is your reason and what is your reference for this? Employing for example ArcGIS on either police or pre-hospital data, researchers can access on hot spot location based on their interest.

- Thank you for this comment. Yes, it is true that geolocation can occcur on both police or pre-hospital data as long as these datasets are inclusive of the information needed to allow a geolocation to be ascertained. Many prehospital prehospital data and police data might not have latitude nor longitude information nor will street addresses exsist with enough certainty to be able to find a coordinate for geolocation. These points were more completely delineated in the second paragraph of the introduction in order to make this reasoning clear.

1. How many registered vehicles are in Kigali? How much are various road length and how about population density as well as demographic information of the resident. The same is needed for Galle, Sri Lanka.

- Further information about Kigali and Galle demographics, population density and road length was included in the study settings.

1. What is categorization of police for injury severity? Are they the same with medical categorization. How about the reliability when different police man evaluate them in difference cases

- Further information about this police categorization was included in the methods. While there is no quality assessment or interrater reliability about this categorization, these categories were further categorized into fatal/grievous and not grievous/ no injuries to minimize these potential limitations.

1. Lines 168-169: This is not well clear, please clarify it.

- Further description was added about lines 168-169 to clarify the questions asked of our high road utilizers and grading the 'dangerous locations.’

1. Line 169: Please kindly explain about the pilot study.

- *Further description of the pilot study goals was included in the methods.*

1. Line 203: When you use sensitivity and specificity you need Gold standard for that. I cannot relay on police as gold standard. Moreover, how can I trust for the survey regarding under-estimation?

- We completely agree that police data is a poor gold standard but there is no obvious alternative. The objective of this project is to identify a possible alternative to police data and, as such, a comparison must be made in order to evaluate this alternative option. We have better described the goals for this project in the introduction and methods.

1. Lines 281: Again what is your Gold standard for judgment?

- Please see prior point for discussion.

1. Lines 282: I don't believe that survey-base is an alternative to be replaced on police. Your recommendation should focus on police quality improvement for their registration, if you agree for that.

- The purpose of the project is to find an alternative for or adjunct to police datasets for researchers and policymakers. This was further delineated in the discussion.

1. Lines 303-304: This is why it is better that you recommend on police register quality improvement since police has legal responsibility for that.

- While having superior quality of police registries would be optimal, in many locations in LMIC, this is not feasible. While we would advocate for improved police records, an alternative or adjunct during this process of improvement is warranted. Further information about this was added to the discussion.

1. Line 309-311: not related

- These two locations were chosen because of their different environments. Rwanda has made incredible strides and improving their road safety while in Sri Lanka there are studies concluding that they have had limited movement on this front.  This statement was made to highlight this difference in locations included and compared.

1. Lines 341-342: citation error

- This error was corrected*.*

1. Lines 359-360: You should think more about this sentence.

- We have discussed and amended this sentence*.*

----------------------------------------------------------------------------------------------------------

**Level of interest:**

An article whose findings are important to those with closely related research interests

**Quality of written English:**

Acceptable

**Statistical review:**

Yes, but I do not feel adequately qualified to assess the statistics.

**Declaration of competing interests:**

I declare that I have no competing of interest

**Reviewer’s Report**

**Title**: High Road Utilizers Surveys Compared to Police Data for Road Traffic Crash Hotspot Localization in Rwanda and Sri Lanka

**Version**: 2 **Date**: 2 September 2015

**Reviewer:** Matthew Kelly

**Reviewer's report:**

This is a very useful methodological paper which addresses a topic of great

importance in low and middle-income countries. The results presented support

the argument for new methods of data collection to identify 'hot spots' for traffic

injury.

The following revisions would be needed though before publication.

----------------------------------------------------------------------------------------------------------

**Minor essential revisions**

1. The methods section is quite limited in terms of the road user surveys. I think

more detail is needed on who the survey participants are. How many potential

participants were approached, how were they approached and by whom? Why

were these particular road users chosen? The method needs to be justified a

little more.

- More detail was added to the methods to further describe how the survey participants were approached and by whom, as well as why they were chosen.

2. Also regarding the data derived from police records more detail is needed.

Have any studies been conducted on the completeness of police record systems

in those settings? In the discussion the authors explain choosing two locations

where "different police reporting infrastructures" (line 288). What are those

infrastructures, how do they differ and what effect might this have on data

reliability?

- More detail about the police was collected. No studies of  these systems in terms of completeness have been conducted that we could find in the literature or per report from police or local stakeholders. The 'different police reporting infrastructures' were better  described in the manuscript.

----------------------------------------------------------------------------------------------------------

Discretionary revisions

1. The Introduction to this paper is very brief. Some of the information presented

in the Discussion, particularly where the settings of the study and the RTC

situation in each setting are described would perhaps be better placed in the

Introduction. This would allow the reader to situate the study more readily.

- We further integrated this information from the discussion into the introduction to allow the reader to better understand the study settings.

----------------------------------------------------------------------------------------------------------

**Level of interest**:

An article of importance in its field

**Quality of written English:**

Acceptable

**Statistical review:**

No, the manuscript does not need to be seen by a statistician.

**Declaration of competing interests:**

I declare that I have no competing interests.
